# Ionic Liquid Mixtures in Task-Specific Applications: Linking Composition, Physicochemical Properties, and Performance

**DOI:** 10.3390/molecules31193559

**Published:** 2026-10-07

**Authors:** Dorota Warmińska, Iwona Cichowska-Kopczyńska

**Affiliations:** 1 Department of Physical Chemistry, Faculty of Chemistry, Gdańsk University of Technology, 80-233 Gdańsk, Poland; dorota.warminska@pg.edu.pl; 2 Department of Process Engineering and Chemical Technology, Faculty of Chemistry, Gdańsk University of Technology, 80-233 Gdańsk, Poland

**Keywords:** ionic liquid mixtures, double-salt ionic liquids, eutectic mixtures, composition–property relationships, thermodynamic non-ideality, functional synergy, task-specific applications

## Abstract

Ionic liquids (ILs) are often described as designer solvents. However, a single cation–anion pair rarely optimizes phase stability, transport properties, and task-specific functions simultaneously. Mixing introduces composition as a continuous design variable, which can suppress crystallization, reorganize coordination and hydrogen-bond networks, redistribute ions on interfaces, and balance viscosity, conductivity, selectivity, and reactivity. This critical review analyses binary and higher-order IL mixtures, including common-ion, reciprocal, double-salt, eutectic, and glass-forming systems, with a focus on studies published from 2018 to 2026, and selected foundational reports. Applications include CO_2_ capture and gas separation, liquid separations, biomass processing, electrochemical energy technologies, thermal management, catalysis, electrospray propulsion, and biological systems. The review connects composition-dependent performance to phase behavior, thermal stability, density and excess volume, transport properties, interfacial organization, polarity, hydrogen bonding, and acid–base descriptors. Special attention is given to counterexamples and to distinguishing eutecticity, thermodynamic non-ideality, and functional synergy. The evidence indicates that the most useful formulation does not necessarily maximize a single property or coincide with the eutectic composition. Its benefit frequently lies in expanding the operating window or balancing multiple requirements. Rational formulation therefore requires composition-resolved measurements, explicit reference states, and multiobjective optimization from molecular structure to process or device performance.

## 1. Introduction

Ionic liquids (ILs) are salts composed entirely of ions and are conventionally defined by melting temperatures below 373.15 K. Their structural diversity arises from the numerous possible cation–anion combinations, as well as the incorporation of task-specific groups. This enables ILs to exhibit tunable physicochemical, solvation, thermal, electrochemical, and interfacial properties. Consequently, ILs are often referred to as “designer solvents” and are employed in separation, catalysis, electrochemical energy technologies, and advanced materials [1]. Optimizing a single cation–anion pair rarely provides the optimal combination of properties required for practical applications. Although strong interactions with gases, solutes, or biopolymers can improve absorption, extraction, or dissolution, they often increase viscosity and limit mass transfer. Electrochemically stable ionic liquids may also exhibit insufficient conductivity or a narrow liquid-temperature range. Therefore, mixing two or more ILs extends the designer-solvent concept by introducing composition as a continuous design variable [2,3]. Adjusting ionic ratios allows for the tuning of phase behavior, viscosity, conductivity, polarity, solvation, interfacial composition, and reactivity without the need to synthesize new IL.

Ionic liquid (IL) mixtures should not be treated as conventional mixtures of molecular solvents. When different salts are combined, the original cation–anion associations may not be preserved. Chatel et al. introduced the concept of double-salt ionic liquids (DSILs), highlighting that multicomponent ionic liquids possess well-defined ionic compositions and that their properties arise from the collective interactions of all constituent ions [4]. Recent studies indicate that, even when a mixture appears ideal on a macroscopic scale, significant variations can occur in local structure, hydrogen bonding, free volume, ion mobility, and interfacial organization [5,6,7,8,9]. Therefore, ideality must be defined for a specific property and reference state rather than assigned to a mixture as a whole.

From an application perspective, the main advantage of IL mixing is often the achievement of a more favorable balance among interdependent requirements, rather than maximizing of a single property. Recent studies demonstrate a shift from empirical formulation toward rational composition design. Double-salt ILs have been screened and optimized for separation processes [10], catalytic media [11], electrochemical interfaces [12], and other task-specific applications [13,14]. Moreover, machine learning and process-design approaches increasingly treat mixture composition as an explicit optimization variable [15,16]. These developments indicate that the most useful formulation may not correspond to the composition with the lowest melting point, highest conductivity, or strongest solute affinity, but rather to the one that provides the best overall performance in the application.

Despite this progress, the literature on IL mixtures remains less systematic than that on pure ionic liquids, and several conceptual ambiguities persist. Specifically, the terms eutecticity, thermodynamic non-ideality, and functional synergy are often used interchangeably, although they describe fundamentally different phenomena. Eutecticity concerns solid–liquid phase equilibrium and a minimum in the liquidus temperature. Thermodynamic non-ideality refers to deviation from a clearly defined ideal reference state, whereas functional synergy should be reserved for cases in which a specified application metric exceeds an appropriate benchmark. A low melting or solidification temperature alone does not demonstrate deep-eutectic behavior, nor does a non-linear composition dependence necessarily indicate enhanced application performance.

In this context, this review critically evaluates binary and higher-order IL mixtures as compositionally engineered media for task-specific applications. It considers common-cation, common-anion, reciprocal, double-salt, eutectic, and glass-forming systems, relating ionic identity and composition to molecular organization, bulk and interfacial properties, and functional performance. The review covers CO_2_ capture and gas separation, liquid separations, metal recovery, chromatography, cellulose and biomass processing, electrochemical energy technologies, thermal management, catalysis, electrospray propulsion, functional materials, and emerging biological applications. Counterexamples are retained to distinguish genuine enhancement from intermediate or diminished performance.

Literature was searched in Scopus, Web of Science, and Google Scholar through August 2026 using combinations of the terms “ionic liquid mixture”, “mixed ionic liquid”, “double-salt ionic liquid”, “eutectic ionic liquid”, and application-specific keywords. Studies published from 2018 to 2026 were prioritized; earlier reports were included only if foundational or if no recent equivalent was available.

## 2. Nomenclature and Classification

The nomenclature classification of ionic liquid mixtures is less straightforward than that of mixtures of neutral molecular solvents. A molecular solvent generally preserves an unambiguous molecular identity after mixing, whereas an ionic liquid is composed of separately mobile cations and anions. As a result, an ionic mixture can be described by the salts used for its preparation, the ionic species present in the liquid, or the minimum number of thermodynamic components required to define its composition. Although these descriptions are related, they are not interchangeable. Niedermeyer et al. distinguished between a constituent, defined as an individual chemical species present in the system, and a thermodynamic component, defined as an independently variable entity required to describe the composition of all phases [3]. For example, a mixture of two ionic liquids prepared from [A][X] and [B][X] contains three ionic constituents (A^+^, B^+^, and X^−^) but only two thermodynamic components, as the electroneutrality constraint prevents independent variation in ionic concentrations. Similarly, a mixture prepared from two parent salts [A][X] and [B][Y] contains four ionic constituents (A^+^, B^+^, X^−^, and Y^−^) but remains a thermodynamically binary system [17]. Table 1 presents the classification of ionic liquid mixtures based on the number of thermodynamic components and the distribution of ionic species. This distinction is particularly significant in application-oriented studies, since properties such as gas solubility, extraction selectivity, catalytic activity, ionic conductivity and interfacial organization are determined by the complete ionic composition rather than solely by the nominal identity of the parent salts. Therefore, both the parent-salt formulation and the resulting ionic composition should be reported when discussing composition–property–performance relationships in ionic liquid mixtures. Chatel et al. further emphasized that the original counterion assignments should not be assumed to persist after mixing [4]. In systems prepared from [A][X] and [B][Y], all energetically accessible cation–anion contacts among A^+^, B^+^, X^−^, and Y^−^ may influence the liquid structure and properties. On this basis, the authors proposed the term double-salt ionic liquid to denote ionic compositions with more than one type of cation or anion that remain liquid below 373 K [4]. The DSIL concept thus highlights the multicomponent ionic nature of the resulting material rather than treating it as a simple blend of preserved parent ion pairs. Structural studies provide additional evidence for this distinction. Molecular dynamics, infrared spectroscopy, Raman spectroscopy, nuclear magnetic resonance, and quantum-chemical investigations demonstrate that while mixtures may retain the broad structural motifs of the pure ILs, they also exhibit composition-dependent changes in local coordination, hydrogen-bonding networks, nanodomain organization, and free-volume distribution [7,18,19,20,21,22]. Liu et al. demonstrated that the formation of composition-dependent mixed-ion complexes is central to interpreting the behavior of four-ion IL mixtures, whereas Bentley et al. related structural reorganization and non-ideal gas-solubility behavior to differences in anion size and hydrogen-bonding basicity [7,23].

In addition to the classification presented in Table 1, it is important to distinguish strict IL–IL mixtures from broader ionic liquid-based formulations that contain non-IL components. In this review, strict IL–IL mixtures refer to systems composed exclusively of two or more ionic liquids, including common-ion, reciprocal, and higher-order mixtures. In contrast, systems containing an ionic liquid with an added inorganic salt, such as a lithium salt or AlCl_3_, are treated as salt-containing ionic-liquid formulations or electrolytes, whereas mixtures of an ionic liquid with a neutral molecular solvent are classified as IL–molecular-solvent systems.

Within strict IL–IL systems, higher-order mixtures are defined as systems containing three or more thermodynamically independent IL components. They should not be confused with binary reciprocal mixtures, which may contain four ionic species while remaining thermodynamically binary. Higher-order formulations may be designed as common-cation, common-anion, or reciprocal systems and provide additional independent composition variables for tuning phase behavior, transport properties, solvation, and other application-relevant characteristics. These extra compositional degrees of freedom facilitate simultaneous optimization of several properties, but they also considerably increase experimental and modeling complexity because phase behavior and physicochemical properties must be described within a multidimensional composition space. Accordingly, systematic data on ternary and higher-order IL–IL mixtures remain considerably less extensive than those available for binary systems [24,25,26].

## 3. Applications of Ionic Liquid Mixtures

### 3.1. CO_2_ Capture and Gas Separation

The development of efficient CO_2_ capture and gas separation technologies is a critical component of global carbon management strategies aimed at mitigating greenhouse gas emissions. Conventional industrial processes primarily rely on amine absorption, such as monoethanolamine (MEA), which exhibits high reactivity and absorption capacity. However, these systems present significant drawbacks, including high energy requirements for regeneration, solvent loss due to volatility, and equipment corrosion. Ionic liquids have emerged as a superior alternative due to their negligible vapor pressure, high thermal and chemical stability, and exceptional structural tunability [27]. These unique properties allow ILs to serve as alternative separation agents, reducing the energy consumption associated with solvent recovery.

CO_2_ absorption in ILs generally proceeds through two distinct mechanisms: physical absorption (physisorption) and chemical absorption (chemisorption). Physical absorption is predominantly governed by the CO_2_ molecules occupying the free volume or cavities within the IL network [28]. It has been observed that in single IL systems, physical absorption increases linearly with pressure according to Henry’s Law at low to moderate pressures, but often exhibits sublinear behavior at high pressures as the available free volume decreases [29]. In contrast, chemisorption occurs when the CO_2_ molecule reacts with specific functional groups in the IL to form chemical species such as carbamates, carbamic acids, or zwitterions [30]. Functionalization of the cation with amino groups or the selection of basic anions like acetate ([OAc]^−^) or heterocyclic aromatic anions can significantly enhance CO_2_ uptake, particularly at low partial pressures [30]. For example, acetate-based ILs such as 1-butyl-3-methylimidazolium acetate ([BMIm][OAc]) have demonstrated exceptionally high absorption capacities due to their chemical interactions with CO_2_. While chemisorption offers high capacity, it is often associated with a dramatic increase in viscosity upon absorption, which can hinder mass transfer rates [27]. Henry’s law constants reported in the latest literature are presented in Table 2.

#### 3.1.1. The Role of IL Constituents: Anions and Cations

The CO_2_ absorption capacity of an IL is primarily determined by its anion species, while the cation plays a secondary yet significant role [28]. Research indicates that fluorinated anions, including bis(trifluoromethylsulfonyl)imide ([TFSI]−) and tetrafluoroborate ([BF_4_]−), provide high CO_2_ solubility [31,32]. This enhancement is attributed to both the large free volume created by these bulky ions and favorable enthalpic interactions between CO_2_ and the fluorine atoms.

The cation structure further modulates separation performance, particularly through the alkyl chain length. Increasing the length of the cation’s alkyl chain generally enhances CO_2_ solubility by increasing the IL’s hydrophobicity and providing more nonpolar surface area for van der Waals interactions [33]. However, this enhancement must be balanced against the resultant increase in viscosity, which can significantly reduce the diffusion and overall absorption kinetics.

Binary ionic liquid mixtures provide an additional design variable beyond cation–anion selection. Rather than assuming that a mixture exhibits a composition-weighted average of the properties of its pure components, gas absorption and transport must be determined experimentally or explicitly modeled. In particular, there is no general theoretical basis for treating gas solubility in mixed ionic liquid solvents as a linear average of solubilities in the two neat ionic liquids [23]. Non-ideal solubility behavior may therefore be exploited to tune carbon dioxide uptake or membrane performance. Moreover, the formulation of binary IL mixtures has emerged as a powerful strategy to fine-tune thermophysical properties, such as reducing viscosity while maintaining or enhancing CO_2_ separation performance. Mixing two ILs often results in non-ideal behavior, which can lead to synergistic effects [34]. For instance, mixtures of [BMIm][OAc] and 1-butyl-3-methylimidazolium tetrafluoroborate [BMIm][BF_4_] combine the high chemical capacity of the acetate anion with the physical solubility of the tetrafluoroborate anion, which can provide the required selectivity [35].

Mixing two ILs may result in non-ideal composition-dependent behavior, which can be exploited to balance gas sorption, selectivity, and transport properties, but does not in itself demonstrate functional synergy [34]. This distinction is particularly relevant to the terminology used in earlier CO_2_-capture studies. For example, Moya et al. described deviations of CO_2_ Henry’s constants from ideal-mixture behavior as potential “synergistic effects” [36].

Molecular dynamics (MD) simulations and experimental studies indicate that structural reorganization in IL mixtures can influence composition-dependent gas-sorption and transport behavior [37]. In many cases, a smaller anion (e.g., [Cl]^−^) may displace a larger, more weakly coordinating anion (e.g., [TFSI]^−^) from favorable hydrogen-bonding sites near the cation [31]. This can modify local packing and free-volume distribution and may therefore affect the accessibility of sites available to CO_2_. Positive excess molar volumes have also been reported for some IL mixtures and may reflect changes in molecular packing; however, excess molar volume is not equivalent to microscopic free volume and should not be interpreted as a direct predictor of CO_2_ uptake. Gas sorption is determined by the combined effects of packing, specific ion–gas interactions, anion basicity, local coordination, and, where relevant, chemical reactions [34].

The importance of composition-specific design was demonstrated by molecular dynamics simulations coupled with Bayesian optimization for the binary system 1-butyl-3-methylimidazolium bis(trifluoromethylsulfonyl)imide ([BMIm][TFSI]) and 1-ethyl-3-methylimidazolium tiocyanate ([EMIm][SCN]). Twelve simulations were sufficient to identify a composition of 0.490 [BMIm][TFSI] and 0.510 [EMIm][SCN] as the calculated optimum for carbon dioxide uptake. This mixture exhibited a calculated CO_2_ partition coefficient of 2.3846 and an estimated CO_2_ absorption of 66%, exceeding the corresponding calculated values for pure [BMIm][TFSI] (partition coefficient 1.77; absorption 60%) and pure [EMIm][SCN] (partition coefficient 1.46; absorption 53%) [27]. These results support the use of systematic composition optimization rather than selection based solely on the performance of pure ILs.

Experimental measurements of the 1-ethyl-3-methylimidazolium tetrafluoroborate ([EMIm][BF_4_])+1-ethyl-3-methylimidazolium bis(trifluoromethylsulfonyl)imide ([EMIm][TFSI]) and 1-ethyl-3-methylimidazolium dicyanamide ([EMIm][DCA])+[EMIm][TFSI] systems confirm that the CO_2_ solubility of IL mixtures may fall away from ideal mixing behavior. At 313.2 K, the Henry’s law constant for CO_2_ in pure [EMIm][BF_4_] was 90.9 bar, whereas the value in pure [EMIm][TFSI] was 44.3 bar. At an equimolar composition, the measured CO_2_ Henry’s law constant was 57.0 bar, corresponding to greater CO_2_ solubility than predicted by either a linear or a log-linear mixture rule [23]. Since lower Henry’s law constants indicate higher solubility under the convention used in that study, this result demonstrates enhanced CO_2_ sorption in the binary blend relative to simple composition-weighted predictions. For [EMIm][DCA]+[EMIm][TFSI] mixtures, the same study identified substantial composition-dependent changes in CO_2_ solubility. At 313.2 K, Henry’s law constants were 81.3 bar for pure [EMIm][DCA] and 44.3 bar for pure [EMIm][TFSI], whereas an equimolar mixture gave a value of 56.5 bar. At 333.2 K, the corresponding values were 131 bar for pure [EMIm][DCA], 58.8 bar for pure [EMIm][TFSI] and 80.2 bar for the equimolar mixture [23]. The observed deviations from both linear and log-linear mixing rules were larger at 333.2 K than at 313.2 K, indicating that temperature can influence the non-ideal behavior of gas solubility in IL mixtures.

Eutectic ionic liquid mixtures offer an additional approach for overcoming limitations associated with the melting temperature of reactive ionic liquids. Tetrabutylfosphonium acetate ([P_4444_][OAc]) has a melting temperature of 331 K, but eutectic mixtures with [P_4444_][Cl] and [P_4444_][Br] exhibited eutectic temperatures of 297 K and 305 K, respectively. Although dilution of the acetate component reduced the contribution of chemisorption at low pressure, enhanced physical absorption partly compensated for this effect. At pressures above 10 bar and 308.2 K, the eutectic mixtures showed CO_2_ absorption capacities comparable to those of pure [P_4444_][OAc] at 343.2 K [30].

#### 3.1.2. Selective Separation: CO_2_/CH_4_ and CO_2_/N_2_

Specific industrial applications, such as natural gas sweetening and flue gas treatment, require high selectivity for CO_2_ over other gases, such as methane (CH_4_) and nitrogen (N_2_). For post-combustion capture and direct air capture, CO_2_/N_2_ selectivity is considered a key parameter. In the [EMIm][BF_4_]+[EMIm][TFSI] mixture, nitrogen was much less soluble than CO_2_. At 313.2 K, Henry’s law constants for N_2_ were 6600 bar in [EMIm][BF_4_] and 1130 bar in [EMIm][TFSI], compared with 90.9 and 44.3 bar, respectively, for CO_2_ in the same pure ionic liquids [23]. The big difference between CO_2_ and N_2_ Henry’s law constants highlights the preferential uptake of carbon dioxide.

However, enhanced CO_2_ sorption in a mixture does not necessarily yield selectivity greater than that of either pure liquid. In the [EMIm][BF_4_]+[EMIm][TFSI] system at 313.2 K, the reported pure gas CO_2_/N_2_ selectivity was 73 for pure [EMIm][BF_4_], 26 for pure [EMIm][TFSI], and 51 for the equimolar mixture [23]. The selectivity value of each mixture composition falls between the selectivities of the two pure ILs. Similarly, in the [EMIm][DCA]+[EMIm][TFSI] system at 313.2 K, CO_2_/N_2_ selectivity ranged from 124 for neat [EMIm][DCA] to 26 for neat [EMIm][TFSI], with a value of 46 reported for the equimolar mixture [23]. These findings demonstrate that higher CO_2_ solubility alone is insufficient to guarantee satisfying CO_2_/N_2_ selectivity.

The temperature influence follows the general rules. The CO_2_/N_2_ selectivity of pure [EMIm][DCA] decreased from 124 at 313.2 K to 42 at 333.2 K, while the selectivity of the equimolar mixture decreased from 46 to 27 over the same temperature range [23]. This trend was attributed to opposite temperature responses: CO_2_ absorption was exothermic and therefore decreased with temperature, whereas N_2_ absorption in [EMIm][DCA] and [EMIm][BF_4_] increased with temperature, indicating endothermic absorption [23].

In natural gas processing, selection of solvents with a high affinity for CO_2_ compared to CH_4_ is crucial. While pure ILs like [BMIm][OAc] show high CO_2_ uptake, their selectivity can decrease in real gas mixtures due to competitive absorption and non-ideality. Interestingly, it was shown by Finotello et al. that small additions (5–10%) of one IL in a [EMIm][BF_4_]+[EMIm][TFSI] system can simultaneously result in an increase in solubility and selectivity, overcoming the typical industrial trade-off between these two parameters [38].

The separation performance of ionic liquid mixtures should be assessed using realistic multicomponent feeds rather than pure-gas data alone. Monte Carlo simulations of [BMIm][Cl]+[BMIm][TFSI] mixtures at 353 K showed that CO_2_ solubility followed an approximately ideal linear mixing rule at pressures below 20 bar, whereas positive deviations from ideality emerged at higher pressures. In contrast, CH_4_ solubility was generally close to the ideal mixing prediction over the investigated composition range. This difference indicates that ionic liquid mixing can affect CO_2_ uptake more strongly than methane uptake, providing opportunities for tuning CO_2_/CH_4_ separation performance [31]. In the same system, the calculated ideal CO_2_/CH_4_ selectivity increased from approximately eight in pure 1-butyl-3-methylimidazolium bis(trifluoromethylsulfonyl)imide ([BMIm][TFSI]) to approximately 12 in pure [BMIm][Cl]. However, mixed-gas simulations also revealed competitive absorption effects. In pure [BMIm][TFSI], the absorption of both CO_2_ and CH_4_ from mixed feeds was lower than the corresponding single-gas absorption, indicating competition for favorable solvation sites. Therefore, separation performance predicted from pure-gas measurements should be validated under representative feed compositions and pressures [31].

Molecular-scale simulations further indicate that ionic liquid mixtures can provide local environments for different gas species. In equimolar [C_n_MIm][BF_4_]+[C_n_MIm][TFSI] mixtures, CH_4_ preferentially localized around the nonpolar alkyl chains of the cations, whereas CO_2_ and SO_2_ preferentially localized around the anions. The [BF_4_]^−^ anion exhibited stronger Lewis acid–Lewis base interactions with CO_2_ and SO_2_ than [TFSI]^−^, indicating that anion composition can modulate gas-specific local interactions [37].

IL mixtures can be used not only as bulk absorbents but also as liquid phases in supported ionic liquid membranes (SILMs). A reported direct-air-capture-oriented system combined an amine-functionalized ionic liquid, N-(2-aminoethyl)ethanolaminium bis(trifluoromethylsulfonyl)imide ([AEEA][TFSI]), with 1-ethyl-3-methylimidazolium acetate ([EMIm][OAc]). In a 10:90 molar mixture, the initial CO_2_ absorption rate at a partial pressure of 40 Pa was reported to be 116 times higher than that of pure [EMIm][OAc] and 370 times higher than that of pure [AEEA][TFSI] [39]. The membrane measurements were performed using a mixed CO_2_/N_2_ feed with a CO_2_ partial pressure of 40 Pa. SILM reached CO_2_ permeability of 20,902 Barrer, compared with 1760 Barrer for pure [EMIm][OAc] and 206 Barrer for pure [AEEA][TFSI] [39]. The highest reported values in the study were obtained for an [AEEA][OAc]+[EMIm][OAc] mixture at 15:85 molar composition, yielding a CO_2_ permeability of 25,983 Barrer and CO_2_/N_2_ selectivity of 10,059 [39].

The design of IL mixtures for gas separation requires balancing equilibrium uptake with mass transport. Viscosity is especially important because it causes mass-transfer limitations in liquid absorbents and membrane phases. In Bentley et al.’s research on the [EMIm][DCA]+[EMIm][TFSI] system, mixture viscosities were adequately described by an Arrhenius-type mixing model, with reported Grunberg–Nissan interaction parameters of 0.17 and 0.02 for the investigated conditions. It was also shown that the excess molar volumes of both [EMIm][BF_4_]+[EMIm][TFSI] and [EMIm][DCA]+[EMIm][TFSI] mixtures were approximately zero, indicating near-ideal volumetric mixing [23].

Molecular simulations further illustrate an uptake–transport trade-off. In simulations of CO_2_ in [BMIm][BF_4_] and [BMIm][OAc], CO_2_ exhibited higher solubility in [BMIm][OAc], whereas its calculated diffusivity was higher in [BMIm][BF_4_]. At 300 K and 1 bar, at CO_2_ concentrations corresponding to the respective solubility limits, the calculated CO_2_ diffusion coefficients were 3.0 × 10^−10^ m^2^ s^−1^ in [BMIm][BF_4_] and 1.5 × 10^−10^ m^2^ s^−1^ in [BMIM][OAc]. The authors related the higher CO_2_ solubility in [BMIm][OAc] to stronger CO_2_–IL association, which was accompanied by lower CO_2_ diffusivity, while the lower viscosity of the [BMIm][BF_4_] system was consistent with faster CO_2_ transport [32].

Consequently, the optimal mixture for a practical separation process should be selected using multiple criteria rather than gas solubility alone. Relevant parameters include CO_2_ capacity, CO_2_/N_2_ or CO_2_/CH_4_ selectivity, viscosity, diffusion coefficients, membrane stability, water influence and regenerability. The available studies demonstrate that IL mixture composition can modify each of these parameters, often nonlinearly, and that the composition maximizing CO_2_ uptake may not maximize selectivity or gas flux.

**Table 2:** Representative IL mixture studies for CO_2_ capture.

IL1	IL2	Mixture Composition Range Defined as Molar Fraction of IL1	Experimental Conditions	Henry Constant /Atm	Reference
[EMIm][TFSI]	[EMIm][BF_4_]	0 ÷ 1	313.15 K, up to ca 1 atm	100 ÷ 50	[38]
	[EMIm][EtSO_4_]	0 ÷ 1	298.2 K, up to ca 16 atm	117.6 ÷ 36.6	[40]
	[BMIm][EtSO_4_]	0 ÷ 1	298.2 K, up to ca 16 atm	85.5 ÷ 36.6	[40]
	[HMIm][Cl]	0.5	298.2 K, up to ca 16 atm	59.1	[36]
	[4MBPy][TFSI]	0.5	298.2 K, up to ca 16 atm	40.5	[36]
	[EMIm][DCA]	0.5	298.2 K, up to ca 16 atm	46.9	[36]
	[EMIm][EtSO_4_]	0.5	298.2 K, up to ca 16 atm	56	[36]
	[BMIm][PF_6_]	0.5	298.2 K, up to ca 16 atm	74.7	[36]
	[OMIm][PF_6_]	0.5	298.2 K, up to ca 16 atm	64.3	[36]
	[BMIm][TFO]	0.5	298.2 K, up to ca 16 atm	72.3	[36]
	[(OH)2Im][TFSI]	0.5	298.2 K, up to ca 16 atm	58.1	[36]
	[EMIm][BF_4_]	0 ÷ 1	313.2 K, up to ca 15 atm	90.9 ÷ 44.3	[23]
	[EMIm][DCA]	0 ÷ 1	313.2 K, up to ca 15 atm	81.3 ÷ 44.3	[23]
[EMIm][BF_4_]	[OMIm][TFSI]	0 ÷ 1	298.15 K, up to ca 6 atm	0.3626 ÷ 6.733	[41]
[BMIm][BF_4_]	[BMIm][OAc]	0 ÷ 1	298.15 K, up to ca 50 atm	6.733 ÷ 0.3626	[42]
	[OMIM][TFSI]	0 ÷ 1	313.15 K, up to ca 6 atm	3.286 ÷ 15.524	[41]
[EPy][EtSO_4_]	[EMIm][TFSI]	0 ÷ 1	298.2 K, up to ca 16 atm	3.286 ÷ 7.868	[43]
[BMIm][OAc]	[BMIm][TCM]	0 ÷ 1	303.15 K, 6.4 ÷ 58.8 atm	49.5 ÷ 77.8	[44]

### 3.2. Separation and Extraction

Ionic liquid mixtures offer more degrees of freedom than neat ILs because ion identity and composition can be adjusted independently. This makes it possible to balance selectivity, extraction capacity, viscosity, phase disengagement, thermal stability, and solvent recovery. However, mixing is not necessarily synergistic. The optimum composition for equilibrium selectivity may differ from that required for rapid mass transfer or economical solvent regeneration.

#### 3.2.1. Hydrocarbon Separation: Solvent Design and Process Evaluation

Aromatic and aliphatic separation is a representative application in which strong affinity to aromatic compounds must be balanced against solvent capacity, viscosity, and mutual solubility with the hydrocarbon phase. Foundational experimental and COSMO-RS studies have shown that mixed-IL distribution ratios and selectivities can be continuously tuned with composition [45,46,47,48]. Recent work by Xie et al. supports these findings for the separation of o-xylene and n-octane [10]. Vilas-Boas et al. found that adding 1-dodecyl-3-methylimidazolium chloride ([C_12_MIm][Cl]) to 1-butyl-3-methylimidazolium chloride ([BMIm][Cl]) increased affinity and capacity for many organic solutes, but it could reduce selectivity [49]. Zambom et al. also observed that an equimolar 1-butyl-3-methylimidazolium hexafluorophosfate ([BMIm][PF_6_])+[BMIm][Cl] mixture exhibited intermediate polarity, although this did not result in a general synergistic improvement in separation performance [50].

Process-level analysis confirms that solvent selectivity alone is insufficient. For [EMIm][TFSI]+1-ethyl-3-methylimidazolium ethyl sulfate ([EMIm][EtSO_4_]), Ding et al. predicted, relative to a sulfolane process, reductions of 19.6–48.7% in energy use, 6.3–27.1% in total annual cost, and 17.8–47.6% in CO_2_ emissions after process optimization [51]. These are model-based projections, not plant-scale measurements. Wang et al. more recently integrated mixed-IL selection with process intensification to recover isopropanol and n-hexane from wastewater [52]. Together, these studies show that the preferred composition may be governed by circulation rate, regeneration duty, and recovery rather than maximum equilibrium selectivity alone.

#### 3.2.2. Extractive Distillation and Azeotrope Breaking

In extractive distillation, an entrainer must modify the relative volatility enough to remove an azeotrope while remaining fluid, non-volatile, chemically stable, and recoverable. Xing et al. measured isobaric VLE for methyl acetate and methanol in the presence of an equimolar mixture [EMIm][Cl]_0.5_[DCA]_0.5_ at 101.3 kPa [53]. This mixed-anion IL increased the relative volatility of methyl acetate and removed the azeotrope at relatively low entrainer loading.

However, mixing does not always lead to better VLE behavior. Di et al. studied tert-butanol and water with mixed [EMIm][DCA]+[EMIm][OAc] entrainer [54]. The mixture removed the azeotrope, but did not work better than the more effective parent IL. The result shows a compromise between the stronger separation effect of acetate and the lower viscosity associated with dicyanamide. Xu et al. incorporated IL mixture composition directly into computer-aided molecular design using COSMO-SAC and a genetic algorithm [55]. This method treated the ionic ratio as a continuous design variable, allowing separation performance and selected solvent properties to be optimized simultaneously. Still, experimental measurements of VLE, viscosity, corrosion, and solvent recovery are needed before the process can be used. Moreover, in interfacially controlled separation processes, preferential ion enrichment may cause the local interfacial composition to differ from the nominal bulk composition. Consequently, wetting and interfacial mass transfer cannot always be interpreted solely from bulk composition (see Section 4.4).

#### 3.2.3. Metal-Ion Extraction

Foundational studies by Katsuta et al. and Tong et al. showed that combining a coordinating or ion-exchange IL with a hydrophobic IL can selectively extract Pd or Pt from chloride media [56,57]. More recently, Shao et al. used [C_8_bet][Br]+[BMIm][TFSI] for Pd(II) extraction from solutions with several metals, reporting Pd separation factors above 10^3^ and recovery above 91% after three extraction–stripping cycles [58]. Their work shows that combining a hydrophilic, metal-binding IL with a hydrophobic IL can give both selective binding and easy phase separation. Chen et al. combined task-specific 1-tetradecyl-3-propylimidazolium bromide ([C_14_PIm][Br]) with hydrophobic 1-octyl-3-methylimidazolium hexafluorophosfate ([OMIm][PF_6_]) for Pt(IV) extraction followed by direct electrodeposition [59]. These examples couple selective coordination to phase formation, but long-term retention of the designed ionic ratio remains a process requirement.

#### 3.2.4. Chromatographic Stationary Phases

The direct use of IL mixtures as chromatographic stationary phases was initially conducted by Baltazar et al., who used [BMIm][Cl]+[BMIm][TFSI mixtures to tune hydrogen-bond basicity and the retention of alcohols and aromatic compounds [60]. Using a stationary phase containing 75% [BMIm]Cl increased the retention of short-chain alcohols by up to 1100% relative to pure [BMIm][TFSI], and altered the elution order of selected analytes. Zhao and Anderson subsequently extended this concept to binary mixtures of polymeric ILs, showing that their composition controlled both chromatographic selectivity and column-bleed temperature [61]. Because these are polymeric phases, they are retained here as boundary rather than core IL–IL evidence.

Inverse gas chromatography has also been used to determine activity coefficients at infinite dilution and derive separation capacities and selectivities for [BMIm][Cl]+[C_12_MIm][Cl] and [BMIm][PF_6_]+[BMIm][Cl] mixtures [49,50]. Although these results provide useful information about separation capacity and selectivity, they mostly show how the mixtures behave as solvents, not how they work as practical stationary phases in analysis.

Recent chromatography research has focused mainly on neat, multicationic, metal-containing, or polymeric IL phases rather than simple IL–IL mixtures. Chromatography is therefore a demonstrated but still insufficiently developed application of composition-resolved IL mixtures.

### 3.3. Cellulose and Biomass Processing

Cellulose dissolution requires disrupting a dense network of intermolecular hydrogen bonds. Ionic liquids containing strongly hydrogen-bonding anions, such as chloride and acetate, are particularly effective due to their interaction with the hydroxyl groups of cellulose. However, cellulose-rich IL solutions can become extremely viscous, severely limiting mass transfer. IL mixtures allow for variation in both the strength of chemical interactions and transport properties.

The benefits of IL mixing for cellulose dissolution are strongly formulation-dependent. Stolarska et al. reported a pronounced positive mixing effect for a 3:7 mol/mol [EMIm][Cl]+[EMIm][OAc] mixture [62]. At 373 K, its maximum apparent cellulose-loading capacity reached 40 g per 100 g of solvent, compared with 12 and 11 g per 100 g for neat [EMIm][Cl] and [EMIm][OAc], respectively. However, polarized-light microscopy revealed anisotropy at the highest loading; this value should therefore be regarded as a maximum apparent loading rather than an equilibrium solubility. Masiutin et al. subsequently demonstrated that such enhancement is not universal [63]. A 60:40 wt% [BMIm][Cl]+[BMIm][OAc] mixture dissolved 18.5 wt% Avicel, slightly more than neat [BMIm][OAc] (17.5 wt%) and substantially more than [BMIm][Cl] (7.1 wt%). In contrast, the corresponding [EMIm][Cl]+[EMIm][OAc] mixture dissolved 23.4 wt%, which was higher than for neat [EMIm][Cl] (18.0 wt%) but lower than for [EMIm][OAc] (25.5 wt%). Rahman et al. examined the composition effect systematically over the full range of [BMIm][Cl]+[BMIm][OAc] mixtures and identified the 2:3 mol/mol system as the most effective formulation [13]. At 373 K, the reported cellulose concentration reached 32.8 wt%, although approximately 40 h was required to attain this value, and the concentrated solution became difficult to mix. In a subsequent study at 363 K, the same formulation produced dissolution extents of 28.7 and 31.3% for hardwood kraft and prehydrolysis kraft pulps, respectively, demonstrating that residual hemicellulose also influences dissolution [64]. Differences in mixture composition, concentration basis, feedstock and dissolution criteria limit direct comparison of these studies. Nevertheless, their results collectively demonstrate that chloride–acetate mixing can enhance cellulose dissolution, but does not necessarily outperform the better parent IL.

The importance of preserving sufficient hydrogen-bond basicity was confirmed by Mqoni et al. [65]. At 353 K, a 4:1 mol/mol [BMIm][OAc]+[BMIm][SCN] mixture dissolved only 2.8 g of cellulose per 100 g of solvent, compared with 10.3 g per 100 g for neat [BMIm][OAc]. Thus, dilution of the active acetate environment with the less effective thiocyanate anion outweighed any potential improvement in transport properties.

For lignocellulosic biomass, dissolution capacity alone is insufficient; delignification and subsequent enzymatic digestibility are more relevant. Yao et al. pretreated pine with a 1:1 wt/wt [EMIm][OAc]+cholinium lysinate ([Ch][Lys]) mixture at 413 K for 3 h, obtaining glucose and xylose yields of 80.1 and 70.5%, respectively [66]. These values were between those obtained with the parent ILs, demonstrating a useful property compromise rather than synergy relative to the best parent. For sorghum, the equimass system cholinium cation, and lysinate and palimitate anions ([Ch][Lys][Pal]) achieved 86.6% delignification and 98.8% glucose release. The same DSIL was sufficiently biocompatible for an aqueous one-pot pretreatment–saccharification–fermentation process.

More recently, Ding et al. combined a Brønsted-acidic SO_3_H-functionalized IL, [HSO_3_BMIm][Cl], with the Lewis-acidic meta-based IL [BMIm][AlCl_4_] for durian-shell fractionation [67]. The pretreated solid gave a 70% glucose yield after 72 h of enzymatic hydrolysis—more than three times that of the untreated biomass—while the recovered lignin retained antioxidant activity. This example extends IL mixing from physical dissolution to reactive fractionation, in which acidity and selectivity are adjusted simultaneously.

IL mixture performance in biomass processing should be assessed using application-specific metrics, including dissolution time, cellulose integrity, viscosity, delignification, enzymatic sugar yield, solvent recovery, and energy demand. Reported capacities are not directly comparable because concentration bases, feedstocks, water contents, residence times, and dissolution criteria differ.

### 3.4. Energy Conversion, Storage, and Thermal Management

#### 3.4.1. Low-Temperature Electrolytes and Batteries

In batteries, IL mixtures must facilitate the transport of electroactive metal ions while remaining compatible with both electrodes. Their performance therefore depends not only on viscosity and conductivity but also on metal-ion coordination, electrochemical stability, interphase formation, and electrode reaction kinetics.

Huang et al. investigated the common-cation N-methyl-N-propylpyrrolidinium bis(trifluoromethylsulfonyl)imide ([MPPyr][TFSI])+N-methyl-N-propylpyrrolidinium bis(fluorosulfonyl)imide ([MPPyr][FSI]) mixture containing a lithium salt [68]. The ternary Li/IL/IL electrolyte improved rate capability relative to the corresponding single-IL electrolytes and slightly improved cycling stability at 298 K. Raman spectroscopy showed that the anion ratio altered Li^+^ coordination, demonstrating that electrolyte mixing changes both bulk transport and the metal-ion solvation shell.

Vadthya et al. extended the strategy to rechargeable aluminum batteries using the eutectic formulation [EMIm][Cl]+[BMIm][Cl]+AlCl_3_ [14]. The electrolyte exhibited a conductivity of approximately 8.3 mS cm^−1^ and enabled reversible capacities of about 20 and 17 mAh g^−1^ at 253 and 233 K, respectively. Because AlCl_3_ is an additional salt and electroactive Lewis acid, this is formulation-level evidence rather than a strict IL–IL mixture. Related eutectic IL-based magnesium electrolytes have been surveyed recently, but the reported systems vary substantially in salt content and cell chemistry [69].

These results show that IL mixing can simultaneously suppress crystallization, modify metal-ion coordination, and enable battery operation over an extended temperature range.

#### 3.4.2. Supercapacitors and Electrode Interfaces

In electrical double-layer capacitors (EDLC), energy is stored mainly through reversible ion adsorption at porous electrode surfaces. Consequently, the performance of IL mixture electrolytes is governed primarily by the accessible liquid range, viscosity, conductivity, electrochemical stability, and matching between ion size and electrode pore structure.

Newell et al. demonstrated that an equimass [EMIm][TFSI]+[MPPyr][TFSI] mixture enabled supercapacitor operation at 3.5 V over a temperature range from approximately 203 to 353 K [70]. Although the formulation was described by the authors as a eutectic ionic liquid mixture, the reported absence of a detectable phase transition down to 203 K primarily demonstrates a strongly extended liquid range and suppression of crystallization. In the absence of a complete equilibrium solid–liquid phase diagram, this observation alone is not sufficient to establish eutecticity in the thermodynamic sense adopted in this review. Pameté et al. subsequently identified [EMIm][FSI]+[EMIm][BF_4_] mixtures with a mole fraction of [EMIm][FSI] ranging 0.5 ÷ 0.6 as promising low-temperature electrolytes [71]. When combined with hierarchical micro–mesoporous carbon electrodes, the equimolar mixture enabled EDLC operation down to 233 K [72].

Building on this binary electrolyte concept, Yambou et al. introduced a third common-cation IL mixture, 1-ethyl-3-methylimidazolium tetracyanoborate ([EMIm][TCB]), to formulate the ternary electrolyte [EMIm][FSI]_0.6_[BF_4_]_0.1_[TCB]_0.3_ [73]. The mixture remained liquid down to its glass transition at approximately 134 K and exhibited a viscosity of 23.6 mPa s and a conductivity of 14.2 mS cm^−1^ at 293 K.

A pronounced composition effect was also reported by Zhao et al. for symmetric activated-carbon cells containing [BMIm][TFSI] and [EMIm][BF_4_] [74]. At 303 K, the specific capacitance reached 178 F g^−1^ at 20 wt%, exceeding the values obtained with either neat IL, but decreased to 141 F g^−1^ at 80 wt%. Because viscosity and conductivity varied almost linearly with composition, the non-monotonic capacitance was attributed mainly to the combined effects of ion–pore size matching and the geometry and packing of [BF_4_]^−^ and [TFSI]^−^ at the electrode surface.

Mixing does not, however, guarantee improved performance. Mahanta et al. found that the [EMIm][BF_4_]_0.5_[BMIm][BF_4_]_05_ system exhibited an electrochemical stability between that of the parent ionic liquids and a reduced specific capacitance [75]. These results show that the optimum electrolyte composition reflects a compromise among capacitance, operating voltage, ion transport, and power output.

Molecular simulations provide further insight into these interfacial effects. Silva and Colherinhas predicted the highest capacitance for an equimolar hydrated [BMIm][OAc]+[BMIm][benzoate] formulation, but this is simulation-based, water-containing evidence rather than a dry IL–IL experiment [76]. Voroshylova et al. likewise showed through MD simulations of [BMIm][PF_6_]+1-butyl-3-methylimidazolium tris(pentafluoroethyl)trifluorophasphate ([BMIm][FAP]) mixtures at Au(100) that size-dependent competition between anions controls interfacial packing and differential capacitance [12]. The surface-composition measurements of Zhai et al. also demonstrated that the interfacial and bulk compositions of IL mixtures may differ [8]. Although the latter measurements concerned a liquid–vacuum interface, they reinforce the need to optimize both bulk composition and the potential-dependent interfacial structure.

#### 3.4.3. Fuel Cells and Dye-Sensitized Solar Cells

Fuel cells differ from batteries because the electroactive fuel and oxidant are supplied continuously from outside the device. Protic IL mixtures are attractive because their hydrogen-bond networks can support both vehicular and proton-hopping transport.

Miran et al. reported a pronounced electrochemical optimum for N,N-diethylmethylammonium hydrogen sulfate ([DEMA][HSO_4_])+[DEMA][TFSI] mixtures [77]. A formulation containing 56 wt% [DEMA][TFSI] produced an open-circuit potential of 1.03 V in an H_2_/O_2_ fuel cell, compared with 0.90 and 0.77 V for the neat [DEMA][HSO_4_] and [DEMA][TFSI], respectively. Spectroscopic and transport measurements indicated anion–proton exchange and proton hopping through the hydrogen-bond network, in addition to vehicular transport. The device-level maximum therefore resulted from coupled proton-transfer, reaction, and transport processes rather than from conductivity alone.

Abdurrokhman and Martinelli subsequently examined 1-ethyl-3-hexylimidazolium trifluoromethanesulfonate ([EHIm][TFO])+[EHIm][TFSI] mixtures as prospective proton-conducting media [78]. Compositions with (x_TFSI_ = 0.3 ÷ 0.8) showed no crystallization within the investigated temperature range while maintaining relatively high conductivity; activation energies of approximately 0.17–0.21 eV were obtained between 333 and 413 K. Although no fuel-cell device was tested, the work demonstrates that mixing can extend the liquid range without substantially compromising proton transport.

#### 3.4.4. Thermal-Energy Storage and Heat-Transfer Fluids

IL mixtures are also being considered for sensible-heat storage and heat transfer because mixing can modify heat capacity, density, liquid range, viscosity, and thermal stability. The relevant performance parameter is thermal-storage density over the intended temperature interval rather than heat capacity alone.

Mora et al. evaluated fifteen equimolar mixtures derived from six octyl-substituted imidazolium ILs containing [BF_4_]^−^, [PF_6_]^−^, or [TFSI]^−^ anions [79]. Several mixtures exhibited heat capacities and thermal-storage densities higher than expected from simple averaging of the parent properties. Gómez et al., however, found the heat capacities of five equimolar mixtures to be close to the corresponding parent averages of their parent ionic liquids, whereas their phase behavior changed qualitatively: crystallization was suppressed, and only glass transitions were detected within the investigated temperature range [80]. Urzúa et al. subsequently evaluated fifteen mixtures of shorter-chain imidazolium ILs and confirmed that selected formulations could provide favorable thermal-storage densities through the combined contributions of heat capacity and density [81].

These studies demonstrate that mixing may improve thermal performance either by increasing volumetric energy-storage density or by widening the usable liquid range. Practical selection must additionally account for viscosity, thermal conductivity, decomposition during prolonged exposure, corrosivity, and stability over repeated heating–cooling cycles.

### 3.5. Other Applications

#### 3.5.1. Catalysis and Reaction Media

Ionic liquid (IL) mixtures serve as solvents, catalysts, or catalyst-stabilizing media. Their catalytic performance is determined by factors such as acid–base character, hydrogen-bonding ability, polarity, viscosity, and ion accessibility.

Chen et al. studied acetate-based DSILs containing [EMIm]^+^, [Ch]^+^, or [N_2222_]^+^ cations in the coupling of propylene oxide with methanol [82]. Mixing allowed the reaction rate to be adjusted beyond the values obtained with some of the neat ILs. For example, [N_2222_]_0.5_[EMIm]_0.5_[OAc] provided a turnover frequency of 379.8 h^−1^, compared with 358.1 h^−1^ for [EMIm][OAc]. McNeice et al. used binary alkoxide ILS [BMPyr][TFSI]_x_[OiPr]_1−x_ for Knoevenagel and aldol condensations, and the most active formulation outperformed pyridine and sodium methoxide [83]. Xiao and Huang demonstrated that two salts, N-methyl-2-pyrrolidium methyl sulfonate ([HNMP][MeSO_3_]) and [BMIm][Cl], both solid at room temperature, formed a persistent liquid upon mixing [84]. This mixture catalyzed fructose dehydration to 5-hydroxymethylfurfural (HMF) at 298 K, with one-hour HMF yields ranging from 24.6% to 73.0% depending on composition. Marullo et al. used an equimolar [BMIm][Cl]+[BMIm][BF_4_] mixture with sulfonic-acid-functionalized imidazolium catalysts [85]. At 333 K, HMF yields of 60 and 30% were obtained from fructose and sucrose, respectively, and the solvent–catalyst system was reused for four cycles without an appreciable loss of yield.

These results show that catalytic performance in the investigated IL mixtures is related to bulk composition and composition-dependent solvent properties, including acidity/basicity, polarity, hydrogen-bonding ability, viscosity, and ion accessibility.

#### 3.5.2. Electrospray Propellants and Optically Active Ionic Materials

Beyond their conventional use as solvents and electrolytes, IL mixtures can function as advanced ionic materials whose performance is governed by mesoscale organization.

In electrospray microthrusters, ions, clusters, or charged droplets are accelerated by an electric field to generate thrust. Wainwright et al. investigated mixtures of ethylammonium nitrate (EAN) with [EMIm][EtSO_4_] or [EMIm][BF_4_] and [EMIm][EtSO_4_] +[EMIm][BF_4_] systems [86,87]. Conductivity showed pronounced negative deviations from linear mixing, leading to overestimation of the predicted emission current and thrust when ideal mixing was assumed. Mass spectrometry also revealed mixed ion clusters in the [EMIm][EtSO_4_]+EAN plume, demonstrating that emission composition cannot be predicted solely from the parent ILs. Zheng et al. studied [EMIm][DCA]+[BMIm][PF_6_] mixtures using molecular dynamics simulations supported by experimental data [88]. While [EMIm][DCA] favored ion emission and [BMIm][PF_6_] predominantly produced droplets, their 50:50 mass mixture exhibited an intermediate ion–droplet regime, improved thrust and specific impulse relative to [BMIm][PF_6_], and a wider operating-voltage range. Thus, IL mixing enables adjustment of emission mode and propulsion performance, but a reliable formulation requires experimentally determined mixture properties rather than ideal-mixing assumptions.

IL mixtures can also serve as optically active soft materials in which composition controls the local environment of the luminophore and mesoscale organization. Abe et al. studied manganese-containing phosphonium mixtures trihexyl(tetra decyl)phosphonium bromide ([P_666,14_][Br]) + [P_666,14_]_2_[MnBr_4_], which emitted green light in the liquid state at room temperature. Small- and wide-angle X-ray scattering confirmed nanoheterogeneity, while emission intensity increased with temperature, reaching a maximum at approximately 403 K [89].

#### 3.5.3. Emerging Biological Applications and Mixture Toxicity

The biological effects of ionic formulations cannot be inferred from the individual components. Andreu et al. reported synergistic antiproliferative activity in binary amino acid-derived imidazolium IL mixtures, with selected formulations reaching nanomolar IC_50_ values [90]; this is the only strict IL–IL example in this subsection. By contrast, the non-additive responses reported for imidazolium ILs with anticancer drugs [90] and for cholinium IL–inorganic-salt mixtures in microalgae [91] are formulation-level evidence. These early results require validation of pharmacokinetics, systemic toxicity, biodegradation, and chronic ecotoxicity, and they do not justify describing IL mixtures as inherently “green”.

## 4. Targeted Property Tuning: From Molecular Organization to Application Performance

**Figure 1:**
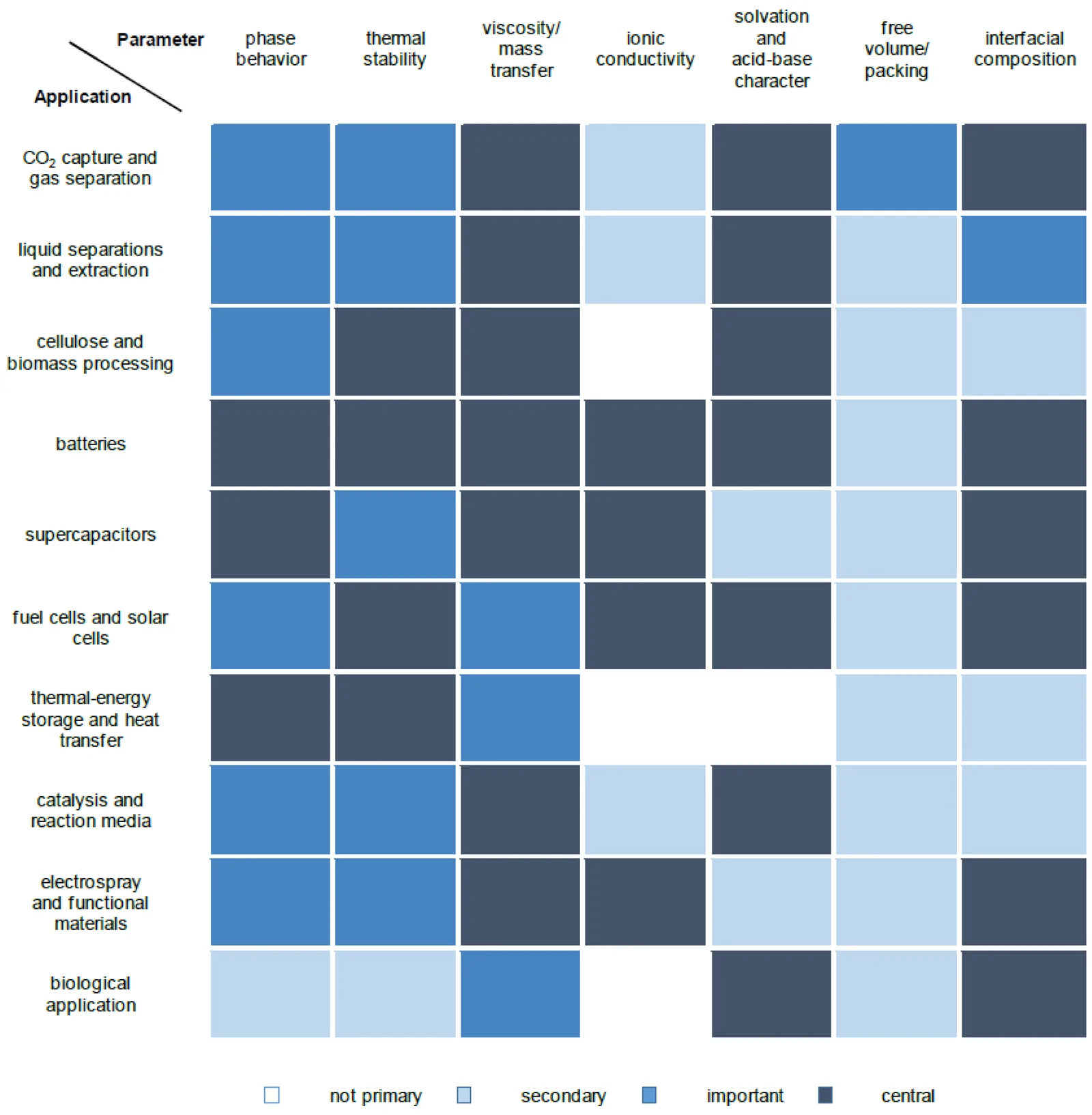
Qualitative application–property map for ionic liquid mixtures. Shading indicates the relative importance of each composition-dependent property within a given application, as synthesized from the literature reviewed. The map is conceptual: categories should not be interpreted as quantitative effect sizes or compared directly between applications.

Section 3 demonstrates that the practical benefits of IL mixing generally emerge from the interplay of several composition-dependent effects. Varying the ionic ratio reorganizes local environments and simultaneously influences phase stability, transport, solvation, and interfacial structure. Nearly ideal density or molar volume may coexist with non-additive phase, transport, or interfacial behavior, whereas even pronounced property deviations do not necessarily improve application performance. This section therefore examines the principal physicochemical factors linking ionic composition to function: phase behavior and thermal stability; density, excess molar volume, and free volume; viscosity and conductivity; surface tension and interfacial composition; and hydrogen bonding, polarity, solvatochromic response, and acid–base descriptors. Figure 1 provides a qualitative map of the relative importance of these factors across the application areas discussed in Section 3.

Prediction of mixture properties requires distinguishing a neat IL from a compositionally variable ionic medium. A neat ionic liquid contains a fixed cation–anion combination, whereas a binary IL mixture introduces composition as an additional independent variable and may contain three ionic species in a common-ion system or four in a reciprocal system. Mixing therefore changes not only the relative abundance of ions but potentially also their local coordination, hydrogen-bonding network, free-volume distribution, nanostructure, and interfacial organization. Consequently, the properties of a binary mixture may be intermediate between those of the parent ILs, may deviate positively or negatively from an additive reference, or may exhibit a composition-specific extremum [6,7,8,9]. The appropriate predictive approach depends on the property and the degree of non-ideality. Simple mixing rules may provide useful first estimates for density or molar volume, whereas viscosity and conductivity are more commonly described using logarithmic or other composition-dependent relationships [6,9,23]. More advanced approaches include the Conductor-like Screening Model for Real Solvents (COSMO-RS) and the Conductor-like Screening Model–Segment Activity Coefficient (COSMO-SAC), quantum-chemistry-based thermodynamic methods used for predicting phase behavior, activity coefficients, solubility, and separation performance [34,48,55]. Molecular dynamics simulations provide molecular-level insight into composition-dependent structure, transport, and interfacial organization [7,12,21,22], while machine learning and other data-driven approaches increasingly enable property prediction and composition optimization [16,27]. Nevertheless, experimental validation remains essential, particularly for strongly non-ideal mixtures.

However, linear or logarithmic mixing rules should be considered reference states rather than universally applicable predictive tools. Current predictive approaches include activity-coefficient and equation-of-state models, COSMO-based methods, molecular simulation, and data-driven models. Thermodynamic models are particularly valuable for phase-equilibrium calculations of ionic liquid (IL) mixtures [92,93,94]. Wu et al. described the solid–liquid equilibrium of six binary protic IL mixtures using the two-suffix Margules equation and applied the resulting activity-coefficient model to calculate their eutectic behavior [92]. For mixtures exhibiting near-ideal liquid behavior, the Schröder–van Laar equation offers a simpler predictive reference based on the melting temperatures and enthalpies of fusion of the pure components. Stolarska et al. demonstrated that the experimentally determined eutectic temperatures and compositions of long-chain [CnMIm][TFSI] mixtures closely matched those predicted under the assumption of near-ideal behavior [94].

The COSMO-RS method was used by Vilas-Boas et al. to reproduce trends in infinite-dilution activity coefficients, capacity, and selectivity for imidazolium-based IL mixtures, supporting their use in solvent screening [49]. Xu et al. applied COSMO-SAC to double-salt ionic liquids. They combined it with a genetic algorithm, enabling both ion identity and mixture composition to be treated as design variables in extractive-distillation solvent optimization [55].

Molecular dynamics simulation was applied by Verma et al. to predict density and conductivity of [EMIm]-based mixtures containing different combinations of [BF_4_]+, [DCA]-, [TFSI]- and [TFO]- [9]. Instead of transferring fixed charges from the pure ILs, the authors derived composition-dependent partial charges from condensed-phase DFT calculations. This approach improved the agreement of predicted properties with the experiment. More recently, Chen et al. combined group-contribution descriptors with ANN, XGBoost, and LightGBM algorithms to predict density, viscosity, heat capacity, and surface tension of binary IL–IL mixtures and integrated these models into solvent and process design [16]. Nevertheless, experimental validation remains essential, particularly for strongly non-ideal mixtures.

### 4.1. Phase Behavior and Thermal Stability

**Figure 2:**
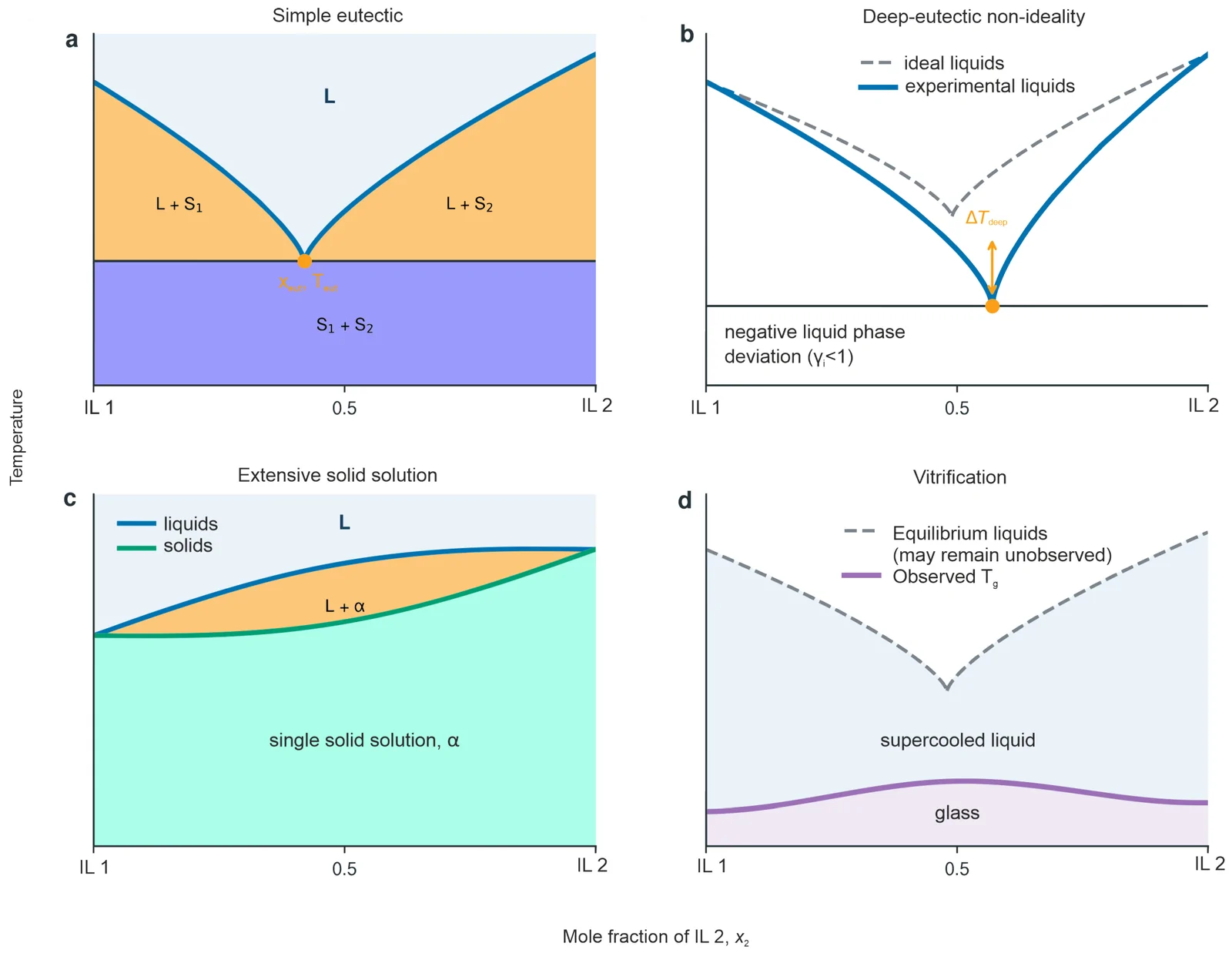
Schematic solid–liquid phase behavior of ionic liquid mixtures: (**a**) simple eutectic formation with negligible solid solubility; (**b**) deep-eutectic behavior, in which the experimental liquidus and eutectic temperature lie below the ideal-mixture prediction; (**c**) extensive solid-solution formation; and (**d**) vitrification, where a glass transition is observed while the equilibrium liquidus may remain experimentally inaccessible. ΔT_deep_ denotes the difference between the ideal and experimental eutectic temperatures. The diagrams are illustrative and are not fitted to a specific system.

For many applications, the ionic medium must remain liquid at the operating temperature. Mixing can lower melting or glass-transition temperatures and suppress crystallization, thereby extending the usable liquid range [71,92,93,94]. Depending on composition and ion structure, IL mixtures may display, as presented schematically in Figure 2, simple eutectic behavior, form solid solutions, or undergo vitrification instead of crystallization [4,17,95,96,97,98]. In some chemically dissimilar systems, liquid–liquid demixing may additionally occur [99].

In IL–IL systems with complete solid–liquid equilibrium data, simple or near-ideal eutectic behavior is frequent. When the liquid approaches ideality, and the components crystallize as separate solid phases, the liquidus branches can be described by the Schröder–van Laar relation. Stolarska et al. found eutectic temperatures of approximately 291–311 K for [C_14_MIm][TFSI]+[C_16_MIm][TFSI], [C_16_MIm][TFSI]+[C_18_MIm][TFSI], and [C_14_MIm][TFSI]+[C_18_MIm][TFSI], close to ideal predictions [81]. Mirarabrazi et al. observed simple eutectics in five common-[PF_6_] subsystems, whereas N-methyl-N-propylpiperidinium hexafluorophosphate ([MPPip][PF_6_])+[MPPyr][PF_6_] formed an extensive solid solution [24]. Elhi et al. identified eutectics in five of eight choline-carboxylate mixtures, with depressions of 13–45 K [100]. A large depression relative to the parent melting points does not by itself prove deep-eutectic non-ideality.

Thermodynamically, a mixture is deep eutectic when its experimental eutectic temperature is lower than the eutectic temperature predicted for an ideal liquid mixture. Such behavior reflects negative deviations from ideality, generally represented by activity coefficients below unity. These deviations indicate that the components are more strongly stabilized in the mixed liquid than in the corresponding ideal mixture. Deep-eutectic behavior has also been demonstrated in mixtures composed exclusively of ILs. Wu et al. investigated six binary mixtures derived from four protic ionic liquids based on [HSO_4_]^−^. All six systems exhibited deep-eutectic behavior, and their solid–liquid equilibrium (SLE) data were best described using a two-suffix Margules model rather than assuming ideality. The largest reported melting-temperature depression, approximately 68.8 K, occurred for 1,3-dimethyl-2-imidazolinone hydrogen sulfonate ([DMIH][HSO_4_])+pyridinium hydrogen sulfonate ([Hpy][HSO_4_]) [92].

The interpretation of phase behavior is complicated by supercooling and slow crystallization. Some mixtures show only a glass transition during DSC measurements even though an equilibrium liquidus may exist [71,78]. Absence of a melting peak therefore does not prove eutecticity or thermodynamic liquidity. Heating rate, thermal history, annealing, and water content should be controlled, and DSC should preferably be supported by X-ray diffraction or polarized-light microscopy.

The eutectic composition is not necessarily the application optimum. Pameté et al. identified [EMIm][FSI]_x_[BF_4_]_1−x_ with x = 0.5 ÷ 0.6 as promising low-temperature electrolytes because they combined low viscosity with high conductivity, not because they coincided with the eutectic point [71]. The eutectic composition should therefore be regarded as a phase-behavior optimum, whereas the best application formulation may lie elsewhere.

Higher-order phase behavior has also been investigated for several all-IL systems. Mirarabrazi et al. determined and modeled the solid–liquid equilibria of a common-cation ternary system and a ternary reciprocal pyridinium-halide system [25]. This approach was subsequently extended to a common-anion quaternary [PF_6_]^−^-based IL system and its ternary subsystems [24]. Bouarab et al. further characterized the phase behavior of a common-anion ternary [PF_6_]-based system [26]. These studies demonstrate that extending IL mixtures beyond binary compositions substantially enlarges the accessible phase space but also increases the complexity of experimental phase mapping and thermodynamic modeling.

Thermal stability is a critical selection criterion for ionic liquid mixtures intended for use at elevated temperatures. In many systems, it is primarily determined by the less stable component [33,101,102]. Yang et al. reported that the decomposition temperatures of binary imidazolium mixtures remained similar to those of the less stable parent IL until the proportion of the more stable component exceeded approximately 0.5 [103]. Most systems decomposed in two stages, with ignition frequently occurring during the first stage. Li et al. demonstrated that prolonged heating at 393 to 513 K reduced the flash points of imidazolium IL mixtures due to the accumulation of combustible decomposition products [104]. Thus, negligible vapor pressure does not guarantee non-flammability under prolonged high-temperature exposure.

Recent results further confirm that thermal decomposition may exhibit molar-ratio-dependent synergistic effects [105]. Lu et al. observed interaction-induced stabilization in [BMIm][BF_4_]_x_[TFO]_1−x_, for which the decomposition-peak temperature reached 703 K at (x_BF4_ = 0.7), compared with 663 and 690 K for the parent ILs [106]. This effect was attributed to preferential formation of stable ion pairs or clusters involving the smaller [BF_4_] anion. Xiao et al. subsequently reported that the 9:1 [BMIm][BF_4_]+[BMIm][TFO] mixture exhibited a T_5%_ of 5.6 K above that of the more stable parent IL [107].

Mixing may decrease, preserve or enhance short-term thermal stability. Consequently, it should be determined experimentally for the actual mixture composition. Moreover, the maximum operating temperature should be based on isothermal decomposition rates, exposure time, atmosphere and the identity and flammability of evolved products rather than solely on the dynamic TGA onset.

### 4.2. Density, Excess Volume, and Free Volume

The usefulness of IL mixtures depends not only on their liquid range but on the manner in which mixing modifies bulk and molecular properties. Density, viscosity, conductivity, polarity and molecular organization are particularly important because they provide the link between composition and application performance.

Most IL mixtures studied at 298.15 K exhibit densities ranging from 1.1 to 1.5 g^.^cm^−3^, with values typically intermediate between those of the parent ILs. Higher densities were observed in systems containing fluorinated anions, particularly [TFSI]^−^. Pamete et al. show that increasing the mole fraction of [EMIm][TFSI] raises the density of its mixtures with [EMIm][BF_4_] and [EMIm][FSI][71]. Similar composition-dependent trends have been reported by Bentley et al. for [EMIm][TFSI] and [EMIm][DCA] and by Miran et al. for [DEMA][HSO_4_] and [DEMA][TFSI] [23,77]. Martins et al. identified analogous patterns in mixtures based on 1-butyl-3-methylimidazolium dimethyl phosphate ([BMIm][DMP]) or [BMIm][TFSI] combined with protic carboxylate ILs, but not in binary [BMIm][DMP]+[BMIm][TFSI] systems [105]. The structure of the cation also influences density: shorter alkyl substituents generally yield denser liquids, as evidenced by the higher density of [EMIm][TFSI] compared to [BMIm][TFSI]. In common-anion systems, imidazolium-based ILs are marginally denser than their pyrrolidinium counterparts [5]. In all cases, density decreases with increasing temperature due to thermal expansion [71,108].

For most IL mixtures, volumetric deviations from ideality, as quantified by the excess molar volume, $V_{m}^{E}$, are remarkably small. Both positive and negative deviations have been reported, with the magnitude and sign depending strongly on the ionic structures involved, packing efficiency, and specific interactions. Annat et al. reported $V_{m}^{E}$ values between approximately −0.25 and 1.5 cm^3^·mol^−1^ for systems containing [MPPyr][TFSI] [109]. Chakraborty et al. observed at 298 K a maximum of about 0.35 cm^3^·mol^−1^ for [EMIm][TFSI]+[BMIm][TFSI] and negative values down to approximately −0.28 cm^3^·mol^−1^ for mixtures containing pyrrolidinium cations [5]. Bentley et al. found [EMIm][BF_4_]+[EMIm][TFSI] and [EMIm][DCA]+[EMIm][TFSI] to be volumetrically ideal within an uncertainty of about 0.2 cm^3^·mol^−1^, while Rodríguez et al. reported only a small positive maximum of about 0.065 cm^3^·mol^−1^ at 298.15 K for [EMIm][BF_4_]+[EMIm][EtSO_4_] [6,23]. Harikumar et al. found that the excess molar volumes of [BMIm][TFSI] mixed with [BMIm][OAc] or with [EMIm][HSO4] followed a sinusoidal trend as composition varied [110]. Buarque et al. reported negative $V_{m}^{E}$ for 2-hydroxyethylammoinum acetate ([2-HEA][OAc]) + bis(2-hydroxyethyl)ammonium acetate ([BHEA][OAc]), which was attributed to more efficient packing. In contrast, positive values were observed for [2-HEA][OAc]+bis(2-hydroxyethyl)ammonium butyrate ([BHEA][Bu]), consistent with steric disruption introduced by the larger butyrate anion [108]. Martins et al. reported more pronounced positive deviations for [BMIm][DMP] mixtures with carboxylate-based protic ILs, with maxima occurring near a protic-IL mole fraction of approximately 0.33 [34]. Clough et al. similarly demonstrated that systems containing chemically dissimilar anions exhibit larger $V_{m}^{E}$, particularly [MeSO_4_]^−^/[TFSI]^−^ and [DMP]^−^/[TFSI]^−^ [111]. Overall, near-zero $V_{m}^{E}$ is typical of structurally similar ILs, whereas larger deviations become more likely when the ions differ substantially in size, polarity, hydrogen-bonding ability, or preferred coordination [112].

Brooks et al. linked excess molar volume to changes in free-volume distribution, although these two quantities are not equivalent [113]. A positive $V_{m}^{E}$ may suggest increased space for gas accommodation, which was a factor in selecting the strongly non-ideal mixtures studied by Martins et al. for CO_2_ capture [34]. However, $V_{m}^{E}$ is not synonymous with free volume, and increased gas uptake cannot be concluded solely from volumetric expansion. Factors such as specific ion–gas interactions, anion basicity, local coordination, and chemical reactions may be equally or more significant. Therefore, a positive excess molar volume is neither a necessary nor a sufficient condition for enhanced gas solubility.

### 4.3. Viscosity and Conductivity

Viscosity is one of the most application-relevant properties of IL mixtures because it directly affects mass transfer, diffusion, and ionic mobility. Unlike density, viscosity does not generally vary linearly with composition, and deviations are usually evaluated relative to a logarithmic mixing rule. Fillion and Brennecke measured the viscosities of 23 IL mixtures and demonstrated that viscosity non-ideality depends not simply on the viscosities of the parent liquids, but on the particular combination of ions present [114]. Mixtures of phosphonium ILs containing aprotic heterocyclic anions ([AHA]) with imidazolium [TFSI]^−^ ILs showed positive deviations from the modified Arrhenius relation, whereas the complementary combinations of imidazolium [AHA] ILs with phosphonium [TFSI]^−^ ILs exhibited negative deviations and consequently, lower-than-expected viscosities. Mixtures sharing a common cation or a common anion were closer to logarithmic additivity. Moreover, four complementary pairs ([C1][A1] + [C2][A2] and [C2][A1] + [C1][A2] mixtures) exhibited identical viscosities at overall mole fractions of 0.50, providing strong evidence that cations and anions exchange freely after mixing rather than retaining the ion-pair identity of the parent ILs [114].

More recent experimental studies confirm that both directions of deviation remain possible. Rodríguez et al. reported clear negative viscosity deviations for [EMIm][BF_4_]+[EMIm][EtSO_4_], which were associated mainly with rearrangement of ionic domains rather than major changes in intermolecular interaction strength [6]. Martins et al. likewise observed negative deviations from the Grunberg–Nissan relation for mixtures of [BMIm][DMP] with carboxylate-based protic ILs, accompanied by the desired reduction in the viscosity of the relatively viscous aprotic ILs; these deviations became more pronounced at lower temperatures [34]. Negative deviations over the investigated composition and temperature ranges were also reported by Buarque et al. for two protic–protic IL systems and by Pamete et al. for [EMIm][FSI]_x_[BF_4_]_1−x_ systems [71,108]. Such behavior is particularly advantageous in applications constrained by mass or charge transport, because the mixture is less viscous than would be expected from logarithmic additivity.

The opposite behavior may also occur. Pameté et al. found that at 293.15 K mixtures of [EMIm][TFSI] with either [EMIm][FSI] or [EMIm][BF_4_] could exhibit viscosities higher than expected and, at some compositions, even higher than those of both parent ILs. Bentley et al. observed only weak deviations for [EMIm][DCA]+[EMIm][TFSI] system, whereas [EMIm][BF_4_]_x_[TFSI]_1−x_ exhibited a slight positive deviation; mixtures containing 50–95 mol% [EMIm][BF_4_] were even slightly more viscous than neat [EMIm][BF_4_] [23].

Overall, there is no universal composition dependence of viscosity in IL mixtures. Structurally similar systems often approach logarithmic additivity, whereas differences in cation structure, anion size, hydrogen-bonding ability, and local organization can produce substantial deviations. For higher-order systems, conventional viscosity mixing laws become less reliable. Bouarab et al. investigated the common-anion ternary [BMPy][PF_6_]+[BMPip][PF_6_]+[BMPyr][PF_6_] system and proposed a viscosity model based on the Gibbs–Adam theory with combined with the Modified Quasichemical Model (MQM). The model showed better predictive performance for ternary viscosity data than the Grunberg–Nissan mixing law with the same number of adjustable parameters [26].

For applications requiring low viscosity, negative viscosity deviations are especially desirable because they offer a means to reduce transport resistance beyond what is expected from simple mixing. Nevertheless, viscosity should be optimized together with other properties, since the composition giving the lowest viscosity does not necessarily provide the best solubility, conductivity, selectivity, or phase behavior.

**Figure 3:**
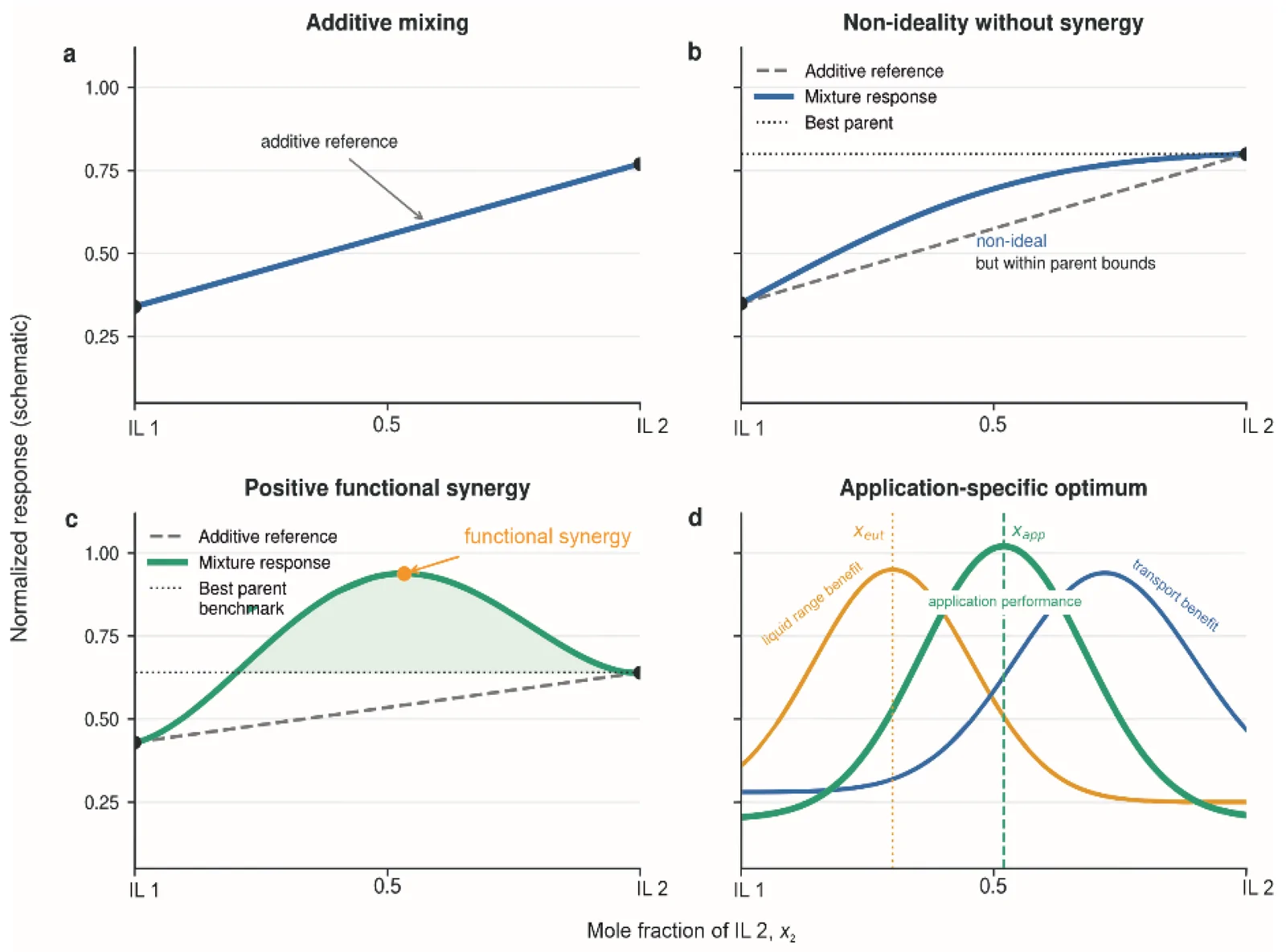
Schematic composition–response relationships in ionic liquid mixtures: (**a**) additive mixing; (**b**) non-additive response without functional synergy; (**c**) positive functional synergy, defined as performance exceeding an explicitly stated best-parent benchmark; and (**d**) a multiobjective application optimum that does not coincide with the eutectic composition or with the optimum of an individual transport property. Curves are illustrative and do not represent a specific dataset.

Composition-dependent responses of IL mixtures can follow several distinct patterns, and non-linearity alone does not establish functional synergy. A mixture may follow an additive reference, deviate from it while remaining within the range defined by the parent ILs, or exceed an explicitly stated best-parent benchmark. Moreover, the composition providing the best application performance may differ from both the eutectic composition and the optimum of an individual transport property. Figure 3 schematically distinguishes these scenarios and illustrates the benchmarks required for interpreting composition–response relationships.

Ionic conductivity is strongly influenced by viscosity, but additional factors such as ion association, charge correlations, specific coordination, and, in protic ionic liquids (ILs), proton-transfer mechanisms, also affect charge transport. Clough et al. found that most of the 11 investigated IL mixture series were well described by a logarithmic conductivity–composition relation. However, no clear correlation was found between the non-ideality parameters derived independently for viscosity and conductivity [111]. Annat et al. similarly observed approximately logarithmic mixing for several systems, whereas structurally distinct mixtures showed more complex behavior; for example, the conductivity of [P_666_,_14_][TFSI]+[MPPyr][TFSI] remained close to that of the phosphonium parent even at x_MPPyr_ = 0.5 [109]. Ning et al. found the conductivities of [OMIm][BF_4_]+[OMIm][Cl], [HMIm][BF_4_]+[HMIm][Cl], and [HMIm][PF_6_]+[HMIm][Cl] to remain between those of the corresponding neat ILs and to vary inversely with viscosity. Their fractional Walden slopes were below unity, indicating appreciable ion association [115]. More pronounced non-additivity was reported by Zhang et al. [116]. The authors observed positive deviations for [DEME]_x_[BMMIm]_1−x_[TFSI], [DEME]_x_[BMMIm]_1−x_[BF_4_], and [BMMIm][TFSI]_x_[BF_4_]_1−x_. For the latter system, conductivity peaked at x = 0.9, exceeding the values of both parent ILs. In contrast, [DEME][TFSI]_x_[BF_4_]_1−x_ showed a negative deviation, with conductivity values remaining between those of the parent compounds. Pameté et al. demonstrated a strong interdependence among conductivity, viscosity, and phase behavior. At 293 K, the conductivities of pure [EMIm][FSI], [EMIm][BF_4_], and [EMIm][TFSI] were 15.26, 12.16, and 7.14 mS cm^−1^, respectively. The mixture [EMIm][FSI]_0.6_[BF_4_]_0.4_ exhibited the highest conductivity among the mixtures at 12.62 mS cm^−1^ and also had the lowest viscosity. Although the room-temperature conductivity of the mixture was lower than that of pure [EMIm][FSI], it maintained a conductivity of 0.46 mS cm^−1^ at 233 K, a temperature at which the pure FSI-based IL was no longer in the liquid state [71]. A similar benefit was observed for the [EHIm][TFO]+[EHIm][TFSI] system by Abdurrokhman and Martinelli [78]. They found only small changes in conductivity with composition; however, the addition of [EHIm][TFSI] strongly suppressed crystallization and widened the liquid-state temperature range. In conclusion, while high conductivity is typically associated with low viscosity, the relationship is not strictly proportional. Positive conductivity deviations are advantageous, although exceeding the conductivity of both parent ILs is not essential. Suppression of crystallization and preservation of ion transport over a wider temperature range may be equally important.

### 4.4. Surface Tension and Interfacial Composition

Surface tension is highly sensitive to the composition of the interfacial layer, which may differ significantly from the bulk composition [117,118]. As a result, IL mixtures can exhibit pronounced deviations from linear mixing behavior, particularly when the components possess markedly different surface affinities [40,119,120,121]. Koller et al. reported that adding small amounts of the fluorinated 3-methyl-1-(3,3,4,4,4-pentafluorobutyl)imidazolium hexafluorophosphate ([PFBMIm][PF_6_]) to [BMIm][PF_6_] led to a substantial decrease in surface tension, consistent with preferential enrichment of the fluorinated component at the interface [122]. Comparable behavior was observed for 1,3-bis(2-(2-methoxyethoxy)ethyl)imidazolium iodide ([(mPEG2)_2_Im][I])+[OMIm][PF_6_], for which Zhai et al. identified a negative deviation of approximately −5.3% at equimolar composition [123]. An even greater effect was found for the common-cation mixture [EMIm][OAc]+[EMIm][TFSI], for which the equimolar mixture showed a −7.7% deviation from linear additivity. Angle-resolved X-ray photoelectron spectroscopy (ARXPS) confirmed preferential enrichment of [TFSI]^−^ at the interface, especially at low bulk concentrations, thereby directly linking macroscopic surface tension to microscopic surface composition [8]. Both positive and negative surface tension deviations have been reported. Buarque et al. observed positive surface tension deviation for [2-HEA][OAc]+[BHEA][OAc], while [2-HEA][OAc]+[BHEA][Bu] showed negative values across the investigated temperature range [108]. Direct evidence for the microscopic origin of such non-linear behavior was also provided by Paap et al., who combined pendant-drop measurements with angle-resolved X-ray photoelectron spectroscopy to correlate surface tension with surface composition [117]. Overall, surface tension cannot be reliably predicted from bulk composition alone, because preferential interfacial segregation may allow even small amounts of a surface-active IL to exert a disproportionately large influence on interfacial properties. This behavior provides an additional strategy for tuning wetting, interfacial mass transfer, and supported-liquid-phase applications without necessarily inducing comparably large changes in bulk properties.

### 4.5. Hydrogen Bonding, Polarity, Solvatochromism, and Acid–Base Descriptors

Polarity in ionic liquid mixtures results from Coulombic interactions, hydrogen bonding, polarizability, dispersion forces, and local ionic organization. Mixing can redistribute cation–anion contacts and alter both the accessibility and residence time of ions around a solute, even when macroscopic properties such as density are nearly additive. Existing studies confirm that hydrogen-bond structure and dynamics may vary non-linearly with composition [22]. Molecular simulations indicate that strongly coordinating anions preferentially occupy the hydrogen-bonding sites of suitable cations and mixing may strengthen specific interactions rather than simply dilute them [20]. Spectroscopic and computational results for [BMIm][TFSI]+[BMPyr][TFSI] showed that imidazolium–anion hydrogen bonding is stronger than pyrrolidinium–anion coordination. Increasing the pyrrolidinium fraction weakened the overall hydrogen-bond network and changed the cis/trans conformational distribution of [TFSI]^−^ [22].

Solvatochromic probes offer an effective method for examining these changes. Reichardt-type dyes yield the normalized polarity parameter, while multiprobe approaches provide the Kamlet–Taft parameters: (α) hydrogen-bond-donor acidity, (β) hydrogen-bond-acceptor basicity, and (π*) dipolarity/polarizability. Thawarkar et al. reported pronounced positive deviations of the polarity parameter for mixtures of the protic ionic liquid [HMIm][OAc] with either [BMIm][TFSI] or [BMPyr][TFSI]. At intermediate compositions, the apparent polarity exceeded that of both parent liquids. This “hyperpolarity” was attributed to hydrogen-bond-mediated restructuring and preferential solvation of the dye, whereas analogous protic–protic mixtures did not exhibit the same synergistic effect [124].

Acid–base descriptors can also be related directly to application performance. In common-acetate double-salt ionic liquids containing [EMIm]^+^, [Ch]^+^, and [N_2222_]^+^, a modified multiparameter relationship incorporating (α), (β), (π*), and Hammett basicity described catalytic activity with (R^2^ > 0.994). Hydrogen-bond basicity was the dominant factor in two mixture families, whereas dipolarity/polarizability became more important in the choline–tetraethylammonium system [82].

Overall, polarity and acid–base parameters should be treated as composition-, temperature-, water-, and probe-dependent characteristics of the complete formulation. No single descriptor fully represents the solvation environment, and parameters obtained for the parent ionic liquids should not be assumed to mix linearly.

## 5. Conclusions and Future Perspective

Ionic liquid (IL) mixtures extend the designer-solvent concept by introducing composition as an additional, continuously adjustable variable. Mixing can suppress crystallization, modify ion association and hydrogen-bond networks, alter preferential coordination, and change interfacial organization. Consequently, the application-optimal composition is not necessarily equimolar or eutectic and generally cannot be predicted by linear interpolation of the properties of the parent ILs.

The most relevant composition–property relationships depend strongly on the intended application. In low-temperature batteries and supercapacitors, for example, crystallization suppression and the preservation of conductivity over a broad temperature range may be more important than achieving the highest conductivity at room temperature. Mixed-anion systems can additionally modify Li^+^ coordination, proton-transfer pathways, and ion packing within electrode pores. In aromatic extraction and extractive distillation, polarity and preferential solvation determine capacity and selectivity, whereas viscosity, phase disengagement, and solvent recovery ultimately govern process feasibility. In cellulose processing, chloride and acetate can cooperatively disrupt cellulose hydrogen bonding, although the outcome depends strongly on their relative proportions and on the cation structure. Replacing part of the acetate with a less basic anion, such as thiocyanate, may improve fluidity but can substantially reduce cellulose dissolution. In catalysis, composition can be used to adjust acidity, basicity, polarity, and the accessibility of active ions. Electrospray propulsion depends on the combined effects of conductivity, viscosity, surface tension, and cluster formation, whereas luminescent mixtures exploit composition-dependent nanoheterogeneity around the emitting species. Biological activity and toxicity must likewise be assessed directly because mixtures may exhibit additive, antagonistic, or synergistic effects.

Gas separation is a particularly important example of the possibilities offered by IL mixtures, as the composition can be used to tune gas solubility, selectivity, phase behavior, viscosity, and transport properties simultaneously. However, the available evidence shows that the effect of mixing is highly system-specific. CO_2_ solubility may show positive deviations from linear mixing at selected compositions and pressures, whereas CO_2_/N_2_ or CO_2_/CH_4_ selectivity may remain intermediate between the values of the pure ILs. Improved CO_2_ uptake alone should therefore not be regarded as evidence of superior separation performance. Instead, capacity, selectivity, permeability, diffusivity, and regeneration behavior should be evaluated together. A central priority for future studies is to move from pure-gas measurements towards realistic mixed-gas testing. Predictions based solely on single-gas Henry’s law constants may therefore fail to represent solvent behavior adequately in biogas upgrading, natural-gas sweetening, pre-combustion capture, or flue-gas treatment. Experiments should consequently employ representative gas mixtures containing CO_2_, N_2_, CH_4_ and, where relevant, acid gases and water vapor, across realistic ranges of pressure, temperature, and feed composition.

A central priority for future studies is to move from pure-gas measurements towards realistic mixed-gas testing. Predictions based solely on single-gas Henry’s law constants may therefore fail to represent solvent behavior adequately in biogas upgrading, natural-gas sweetening, pre-combustion capture, or flue-gas treatment. Experiments should consequently employ representative gas mixtures containing CO_2_, N_2_, CH_4_ and, where relevant, other feed components such as acid gases and water vapor, across realistic ranges of pressure, temperature, and composition. Because the effects of water were not systematically evaluated in the studies reviewed here, no general conclusions regarding its influence on IL mixture performance can be drawn. Nevertheless, for applications involving humid gas streams, water content should be reported and controlled so that its possible influence on phase behavior, viscosity, gas sorption, and transport can be distinguished from the intrinsic effects of IL mixture composition.

The trade-off between reactivity and mass transfer likewise requires systematic consideration. Basic, acetate-containing, amino-functionalized, or otherwise reactive components can enhance CO_2_ affinity through chemisorption, but may simultaneously increase viscosity, slow transport, complicate regeneration, or introduce absorption–desorption hysteresis. Less reactive components, in contrast, may improve fluidity and favor physical sorption, particularly at elevated pressures. For these reasons, future solvent development should rely on multi-objective optimization rather than maximizing a single equilibrium property. Relevant objectives include CO_2_ capacity at the intended partial pressure, selectivity against relevant co-gases, absorption and desorption kinetics, viscosity, diffusivity, thermal and oxidative stability, susceptibility to water, liquid-phase stability, membrane retention where supported ionic liquid membranes are used, and the energy required for solvent regeneration.

Molecular-level characterization should be integrated with macroscopic separation measurements. Combining spectroscopic methods, scattering techniques, molecular simulations, and gas-sorption experiments would help establish direct structure–property relationships relevant to gas separation.

The development of reliable machine learning models for IL mixtures is still limited by the amount and consistency of available experimental data. Many datasets cover only a few compositions, narrow temperature or pressure ranges, or a limited number of IL families. Future studies should therefore report the full ionic composition, several mixture compositions including the pure-component endpoints, temperature, pressure, water content, purity, and experimental uncertainty. It would also be useful to measure several relevant properties for the same compositions under the same conditions, for example density, viscosity, conductivity, phase behavior, solubility, selectivity, and application performance. Reporting both high-performing and poor-performing compositions is important to avoid biased datasets. More standardized and machine-readable data would improve model training, validation, and multiobjective optimization of IL mixtures.

## Figures and Tables

**Table 1:** Recommended classification and terminology for ionic liquid mixture.

Recommended Term	Ionic Species	Typical Parent-Salt Formulation	Number of Thermodynamic Components
Common-cation binary IL mixture	A^+^, X^−^, Y^−^	[A][X]+[A][Y]	2
Common-anion binary IL mixture	A^+^, B^+^, X^−^	[A][X]+[B][X]	2
Binary reciprocal mixture	A^+^, B^+^, X^−^, Y^−^	[A][X]+[B][Y] or [A][Y]+[B][X]]	2
Ternary reciprocal mixture	A^+^, B^+^, X^−^, Y^−^	Any three independent salts selected from [A][X], [A][Y], [B][X], and [B][Y], e.g., [A][X]+[B][X]+[B][Y]	3
Common-cation ternary IL mixture	A^+^, X^−^, Y^−^, Z^−^	[A][X]+[A][Y]+[A][Z]	3
Common-anion ternary IL mixture	A^+^, B^+^, C^+^, X^−^	[A][X]+[B][X]+[C][X]	3

## Data Availability

No new primary data were generated in this study. The data analyzed in this review were derived from previously published studies cited in the reference list.

## Abbreviations

Abbreviation	Meaning
CATION ABBREVIATIONS	
[(mPEG_2_)_2_Im]^+^	1,3-bis(2-(2-methoxyethoxy)ethyl)imidazolium
[2-HEA]^+^	2-hydroxyethylammonium
[4MBPy]^+^	1-butyl-4-methylpyridinium
[AEEA]^+^	N-(2-aminoethyl)ethanolaminium
[BHEA]^+^	bis(2-hydroxyethyl)ammonium
[BMIm]^+^	1-butyl-3-methylimidazolium
[(OH)2Im]^+^	bis(hydroxy)imidazolium
[BMPyr]^+^	N-butyl-N-methylpyrrolidinium
[BMPip]^+^	N-butyl-N-methylpiperidinium
[EPy]^+^	1-ethylpyridinium
[EHIm]^+^	1-ethyl-3-hexylimidazolium
[C_8_bet]^+^	octyl betaine ester cation
[C_14_PIm]^+^	1-tetradecyl-3-propylimidazolium
[Ch]^+^	cholinium
[C_n_MIm]^+^	1-alkyl-3-methylimidazolium; n denotes the number of carbon atoms in the alkyl chain
[DEMA]^+^	N,N-diethylmethylammonium
[DEME]^+^	N,N-diethyl-N-methyl-N-(2-methoxyethyl)ammonium
[DMIH]^+^	protonated 1,3-dimethyl-2-imidazolidinone
[EMIm]^+^	1-ethyl-3-methylimidazolium
[HMIm]^+^	1-hexyl-3-methylimidazolium
[C_12_MIm]^+^	1-dodecyl-3-methylimidazolium
[HNMP]^+^	N-methyl-2-pyrrolidonium
[Hpy]^+^	pyridinium
[N_2222_]^+^	tetraethylammonium
[P_4444_]^+^	tetrabutylphosphonium
[OMIm]^+^	1-octyl-3-methylimidazolium
[P_666,14_]^+^	trihexyl(tetradecyl)phosphonium
[PFBMIm]^+^	3-methyl-1-(3,3,4,4,4-pentafluorobutyl)imidazolium
[PMPip]^+^	N-methyl-N-propylpiperidinium
[MPPyr]^+^	N-methyl-N-propylpyrrolidinium
ANION ABBREVIATIONS	
[AlCl_4_]^−^	tetrachloroaluminate
[BF_4_]^−^	tetrafluoroborate
[Br]^−^	bromide
[Bu]^−^	butyrate (butanoate)
[TCM]^−^	tricyanomethanide
[TCB]^−^	tetracyanoborate
[MeSO_3_]^−^	methanesulfonate (mesylate)
[Cl]^−^	chloride
[DCA]^−^	dicyanamide
[DMP]^−^	dimethyl phosphate
[FAP]^−^	tris(pentafluoroethyl)trifluorophosphate
[EtSO_4_]^−^	ethyl sulfate
[FSI]^−^	bis(fluorosulfonyl)imide
[HSO_4_]^−^	hydrogen sulfate (bisulfate)
[I]^−^	iodide
[Lys]^−^	lysinate
[MeSO_4_]^−^	methyl sulfate
[MnBr_4_]^2−^	tetrabromidomanganate(II)
[OAc]^−^	acetate
[OiPr]^−^	isopropoxide
[Pal]^−^	palmitate
[PF_6_]^−^	hexafluorophosphate
[SCN]^−^	thiocyanate
[TFO]^−^	trifluoromethanesulfonate (triflate)
[TFSI]]^−^	bis(trifluoromethanesulfonyl)imide
